# Clinical Outcomes of Interlaminar Percutaneous Endoscopic Decompression for Degenerative Lumbar Spondylolisthesis with Spinal Stenosis

**DOI:** 10.3390/brainsci11010083

**Published:** 2021-01-10

**Authors:** Pornpavit Sriphirom, Chaiyaporn Siramanakul, Preewut Chaipanha, Chalit Saepoo

**Affiliations:** 1 Department of Orthopaedic Surgery, Rajavithi Hospital, Rangsit University, Bangkok 10400, Thailand; pornpavit@yahoo.com (P.S.); Preenarak@gmail.com (P.C.); bell_ass@hotmail.com (C.S.); 2 Department of Orthopaedic Surgery, Paolo Memorial Hospital Phaholyothin Medical Center, Bangkok 10400, Thailand

**Keywords:** degenerative spondylolisthesis, interlaminar percutaneous endoscopic decompression, spinal stenosis

## Abstract

The use of traditional open decompression alone in degenerative spondylolisthesis can lead to the development of postoperative spinal instability, whereas percutaneous endoscopic decompression can preserve the attachment of intervertebral muscles, facet joint capsules, and ligaments that stabilize the spine. The study’s aim was to determine clinical as well as radiologic outcomes associated with interlaminar percutaneous endoscopic decompression in patients with stable degenerative spondylolisthesis. For this study, 28 patients with stable degenerative spondylolisthesis who underwent percutaneous endoscopic decompression were enrolled. The clinical outcomes in terms of the visual analogue scale (VAS) and Oswestry disability index (ODI) were evaluated. Radiologic outcomes were determined by measuring the ratio of disc height and the vertebral slippage percentage using lateral standing radiographs. The average follow-up period was 25.24 months. VAS and ODI were significantly improved at the final follow-up. In terms of ratio of disc height and vertebral slippage percentage found no significant difference between the preoperative and postoperative periods. One patient underwent further caudal epidural steroid injection. One patient underwent fusion because their radicular pain did not improve. Interlaminar percutaneous endoscopic decompression is an effective procedure with favorable outcomes in selected patients with stable degenerative spondylolisthesis.

## 1. Introduction

Degenerative lumbar spondylolisthesis is described as a vertebra experiences a displacement in the anterior relative to vertebra below it. The process of degenerative changes occurring in intervertebral disc and facet joints results in vertebral slippage and spinal stenosis [1]. Degenerative lumbar spondylolisthesis often affects levels L3–4 and L4–5 and presents with spinal stenosis or spinal stability. These pathologies can induce back pain, claudication, or radiculopathy. Matsunaga et al. described the natural history of degenerative lumbar spondylolisthesis as progression of degenerative changes going through phases that first include a state of instability and then a state of stability [2]. Although nonoperative management is the first treatment option, many cases require surgery after conservative treatment failure. The target of surgery is decompression of the relevant neural structure for patients with spinal stenosis and fusion for patients with spinal instability. However, there is an open debate regarding how surgical treatment options between decompression without fusion compare to the combination of decompression and fusion for patients with degenerative lumbar spondylolisthesis without dynamic spinal instability. Findings from meta-analyses and systematic reviews indicated that there is modest evidence that combination of decompression and fusion show better outcomes [3,4,5,6,7]. Traditional open decompression alone can lead to the development of postoperative spinal instability, which adversely effects clinical outcomes compared with fusion [8]. Currently, some studies have confirmed the effectiveness of using full endoscopic spinal surgery as a treatment option for lumbar spinal stenosis [9]. Endoscopic spinal surgery procedure requires a minimal incision and substantially less damage to the soft tissue, as well as protection of facet joints and posterior ligaments, which may result in maintaining stability of the vertebral segment compared with conventional open surgery.

Our aims of study were to prospectively determine the clinical outcomes of patients with degenerative spondylolisthesis with spinal stenosis who underwent fully endoscopic interlaminar decompression and the postoperative progression of the slip and disc height ratio.

## 2. Materials and Methods

From January 2015 to April 2020, patients with degenerative spondylolisthesis with spinal stenosis were recruited for the study. To be included in the study, a patient had to have either neurogenic claudication or radiculopathy symptoms accompanied by spinal stenosis from an MRI scan. For such patients, there also had to be recorded that their conservative treatment had failed for a period of not less than three months. Patients with lumbar scoliosis (coronal Cobb angle ≥10°) were included in this study. The exclusion criteria were patients who had previously undergone any form of spinal surgery, more than two levels of spinal stenosis, mainly back pain symptoms, and dynamic instability (>4 mm motion on flexion/extension radiographs). A total of 42 patients underwent interlaminar percutaneous endoscopic decompression (PED) at L3–4, L4–5, or L5-S1 at the Rajavithi Hospital by a single surgeon (P.S.). A total of 13 patients were excluded because they did not respond to the postoperative follow-up request by phone. One patient was excluded from analysis because she had only mild numbness in her leg and did not suffer from pain or disability. Finally, 28 patients were analyzed in this study.

The patient demographics, operative details, operative times, follow-up periods, and complications associated with endoscopic procedures were retrospectively analyzed. The radiographs and MRI images of patients were recorded, including Cobb angle for lumbar scoliosis, spondylolisthesis grade using the system of classification by Meyerding [10], and vertebral slippage percentage (% slip) preoperatively and postoperatively. The slippage percentage was calculated from anteroposterior displacement of L4 (L3) over L5 (L4) divided by anteroposterior diameter of L5 (L4) on lateral radiographs. The disc height was evaluated on lateral radiographs at the midpoint of interspace between upper and lower endplates. The width of the upper endplate of lower vertebrae was measured. The ratio of disc height was calculated from disc height at each operative level divided by width of upper endplate of the lower vertebral body.

To quantify clinical outcomes, the visual analogue scale (VAS; full score = 10) was utilized to measure neurologic pain outcomes and Oswestry disability index (ODI) scores [11] were used to determine impairment. Scores for ODI range from 0 to 100 with the highest level of impairment represented with 100. These clinical outcome parameters were evaluated twice, first at the preoperative stage and as a final follow-up after the operation. Recovery rate of the VAS and ODI were determined. Calculations for the scores used recovery rate of VAS = 100 × (preoperative VAS − postoperative VAS)/preoperative VAS, and recovery rate of ODI = 100 × (preoperative ODI − postoperative ODI)/preoperative ODI.

### 2.1. Surgical Technique

The percutaneous endoscopic decompression (PED) for spinal stenosis procedure is performed with the patient under general or epidural anesthesia, depending on the anesthesiologist’s preference. The patient is placed in the prone position as their hip and knee are flexed. The initial target point is the lateral edge of the interlaminar window on the symptomatic side and level under fluoroscopic guidance. The skin incision of 10 mm is created through subcutaneous tissue and thoracolumbar fascia. A blunt dilator is inserted through the incision toward the inferior border of the upper lamina, and the paraspinal muscles are dissected from the bone of lamina. After dilation, a cannula having a bevel is placed on the lamina surface as the endoscope is presented. Inferior border of upper lamina, ligamentum flavum, and medial aspect of the facet joint are identified using a bipolar electrode and micropunches.

### 2.2. Decompression

**Figure 1:**
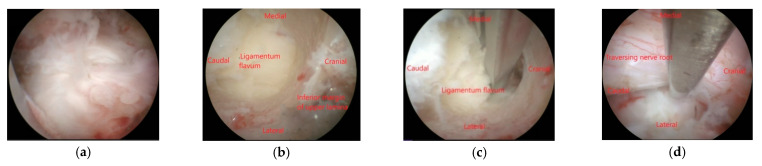
(**a**) Facet joint capsule must be preserved. (**b**) Laminotomy using endoscopic burr starting from inferior border of upper lamina. (**c**) Ligamentum flavum removed with Kerrison rongeur. (**d**) Lateral border of traversing nerve root is visualized.

**Figure 2:**
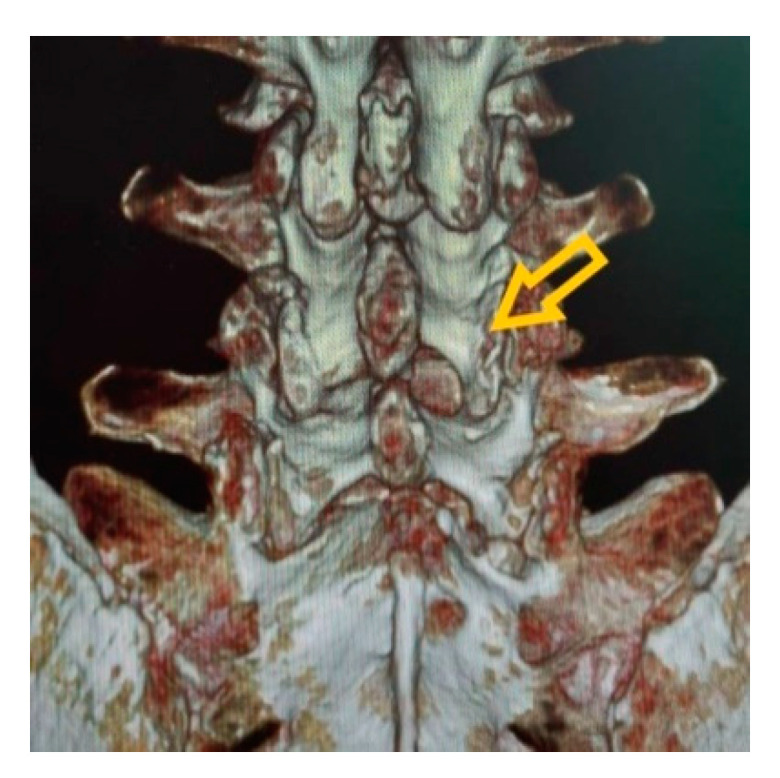
Postoperative CT scan after interlaminar percutaneous endoscopic decompression (PED). Yellow arrow indicates area of laminotomy.

Endoscopic laminotomy is performed by starting from the inferior border of the upper lamina to the medial border of the inferior articular process of the ipsilateral facet joint using the endoscopic burr until the edge of the ligamentum flavum is exposed (Figure 1b). Decompression can proceed, including cranial and caudal laminotomy and removal of ligamentum flavum (Figure 1c). Medial facetectomy should be performed less than 50% by undercutting. The facet capsule and ligament must be preserved (Figure 1a). The thecal sac is identified and the lateral border of the traversing nerve root should be visualized after decompression (Figure 1d). The traversing nerve root should be examined to confirm adequate decompression. Area of laminotomy is shown in Figure 2.

In the case of bilateral stenosis, further decompression of the contralateral side is required after ipsilateral decompression. The endoscope is directed toward the contralateral side above the dural sac. Ligamentum flavum removal and laminotomy are performed by an undercutting technique until the medial aspect of contralateral side can be reached.

### 2.3. Statistical Analysis

Collected data were computed as mean and ± standard deviation. The preoperative and postoperative ODI and percentage of slip were matched using paired-sample test. The comparison of pre- and postoperative VAS and disc height ratio was analyzed using the Wilcoxon signed ranks test. Statistical differences were considered when *p* < 0.05.

## 3. Results

The study had a total of 28 patients, 6 men and 22 women having an average age of 63.92 years. The average follow-up period was 25.24 months. With regard to presence of deformity, three patients had spondylolisthesis with coexisting scoliosis. Patients with scoliosis presented with an average Cobb angle of 12.33° ± 2.08°. Most slippage was grade 1. Only two patients presented with grade 2 slippage (Table 1).

The level of surgery, type of anesthesia, and operative times are shown in Table 2. In all, 64.3% of patients underwent unilateral L4–5 decompression, 21.4% underwent bilateral L4–5 decompression, and 14.3% underwent two-level decompression. The majority of anesthesia was general anesthesia, and 17.9% of patients received epidural anesthesia. The average operative time was 135.47 ± 43.77 min.

The mean of vertebral slippage percentage (% slip) was 14.99 ± 7.39% (Table 3). The preoperative VAS and ODI scores were 9.35 ± 0.78 and 55.79 ± 16.75, respectively (Table 3). The patient-reported outcomes at the final follow-up are reported in Table 3. Patients in the series experienced significant improvement in VAS and ODI scores compared to preoperative values.

Moreover, 67.9 and 71.4% of patients experienced more than 50% improvement of VAS and ODI scores, respectively (Table 4). There was no significant difference in % slip and disc height ratio between preoperative and postoperative periods (Table 3).

Among the 28 patients who underwent interlaminar PED, two patients needed further procedures. One patient had buttock pain after surgery and received a caudal epidural steroid injection. One patient did not have improved radicular pain after endoscopic surgery and underwent further fusion surgery (Table 5).

## 4. Discussion

The process of degenerative spondylolisthesis begins with a phase of facet and disc destruction, leading to translation of the vertebral body on the other side, after that the degenerative changes take place to cause stabilization of the intervertebral level, just as is the cascade of degenerative disc disease introduced by Kirkady–Willis [2,12]. The potential findings of a restabilizing phase that can be identified with radiographs are facet sclerosis, disc space narrowing, peridiscal osteophytes, and endplate sclerosis [2,13]. The degenerative process may also cause either displaced spinal canal or spinal stenosis.

The aims of surgical procedure in degenerative spondylolisthesis consist of decompression of the spinal canal stenosis and stabilize unstable spondylolisthesis with fusion. Meanwhile, the benefit of fusion for low-grade or stable spondylolisthesis has been controversial. Ghogawala et al. conducted a randomized study involving 66 patients with lumbar spondylolisthesis to compare between decompression alone and decompression combined with fusion procedure. Findings showed that patients who underwent decompression in combination with fusion provided higher SF-36 scores and lower reoperation rate than the decompression without fusion group. [8]. Nevertheless, Inose et al. found no difference in clinical outcomes in terms of Japanese Orthopedic Association (JOA) scores and VAS between decompression alone, decompression with fusion, or decompression with stabilization in patients with low-grade spondylolisthesis [14]. However, clinical symptoms and anatomical pathology may indicate options of surgical procedures. Several studies indicated that two main criteria for selecting decompression alone in the degenerative lumbar spondylolisthesis population were (1) predominantly radicular and neurogenic claudication symptoms, and (2) grade 1 spondylolisthesis with <3–5 mm on dynamic radiographs [15,16,17]. The conventional laminectomy procedure may induce postoperative lumbar instability in spondylolisthesis patients. The reason for this is that the paraspinal muscles and other parts such as facet joints and spinous process are dissected sequentially. A study by Epstein found that 31.4% of spondylolisthesis patients developed further slip from grade 1 to grade 2 after two-year follow-up [18]. From this cause, minimally invasive spinal surgery has played an important role in preserving posterior structures of the lumbar spine. Patients with lumbar spondylolisthesis undergoing microendoscopic laminotomy via interlaminar approach have reported 69% good/excellent results, and the average slippage percentage was not significantly different between preoperative and final follow-up [19].

Hence, there is currently strong evidence to support the use of full endoscopic surgery as an alternative intervention for operations in the spine [20]. The most advanced procedure for spinal endoscopy today is using lumbar spinal stenosis decompression [21]. Recently a meta-analysis of fully endoscopic decompression for spinal stenosis via the interlaminar approach found improvement of postoperative ODI scores by 41.71, as well as improvement of VAS leg and back pain scores by 5.95 and 4.22, respectively [22]. Moreover, the conventional decompression and fusion may increase risk of perioperative complication in elderly patients or comorbidity patients. Hence, in these cases the full-endoscopic procedures have their best indication as the first choice for symptom control without destabilizing the spine.

Therefore, we hypothesized that interlaminar percutaneous endoscopic decompression for spinal stenosis in degenerative spondylolisthesis would provide optimum clinical outcomes and maintenance of postoperative spinal stability. In this prospective study, patients with stable degenerative spondylolisthesis underwent PED. This study recorded mean VAS and ODI scores at 25-month follow-up were significantly improved from those obtained before surgery. Moreover, 67.8 and 71% of patients experienced more than a 50% improvement rate in VAS and ODI scores, respectively. Our finding is consistent with that of Youn et al., who reported on 23 patients with spondylolisthesis and spinal stenosis who underwent endoscopic posterior decompression under local anesthesia and were followed up for two years. The study found significant improvements in ODI scores from 46.1 to 23.6 and VAS scores from 75.4 to 23.5 [23].

In this study, no significant difference was found in postoperative slippage percentage (% slip) compared to the preoperative slippage percentage. Youn et al. also reported no significant change in the values of lumbar lordosis, disc-wedge angle, or slip percentage after two years follow-up period [23]. PED can preserve the attachment of intervertebral muscles, facet joint capsules, and ligaments, which prevent destabilizing the spine. Hence, it can also assist in postoperative strengthening of the back muscles and maintaining the motion segment stability.

One interesting finding is a slight decrease of the disc height ratio at the last follow-up, which indicates a collapse of disc space and the spine changing into the restabilization phase. However, the no significant difference was found, due to the limited time for follow-up, which was two years. Minamide et al. found that 35% of patients with unstable degenerative spondylolisthesis who underwent microendoscopic laminotomy had disc space collapse during a three-year postoperative period [19].

Of 28 patients taken through PED, one patient needed caudal epidural steroid injection due to buttock pain after surgery, and pain was finally improved, and one patient underwent fusion surgery because their radicular symptoms did not improve. Therefore, a 3.5% rate of reoperation fusion in the current study is not different from what previous authors have found [23]. In degenerative spondylolisthesis, the notion of a full-endoscopic procedure focuses on the anatomical pathologies responsible for nerve root compression. Transforaminal endoscopic foraminoplasty should be considered for patients who suffer radicular symptoms that result from the narrowing of the intervertebral foramen in degenerative spondylolisthesis. Jasper et al. performed transforaminal endoscopic discectomy with foraminoplasty in 21 patients with degenerative spondylolisthesis. The study reported 71.9% good results as defined by MacNab. The average VAS score was reduced from 8.48 to 2.30 at postoperative one year [24].

There were some limitations with the study. The first of these limitations was the small number of cases used, which made it impossible to have a control group. The small number of cases was, however, caused by the fact that as many as fourteen patients had to be excluded from the study, either because they did not meet the inclusion criteria or they failed to respond to follow-up calls. Due to the fact that the follow-up period allocated in this study was short, it is recommended that future studies that expand on this should use a larger patient size and also increase their follow-up periods.

## 5. Conclusions

Interlaminar percutaneous endoscopic decompression is an effective procedure with favorable results when used for patients who have stable degenerative spondylolisthesis and concurrent stenosis. Moreover, the slippage rate did not progress two years after the operation. The implication of this is that preserving vertebral structures during the use of interlaminar PED permits degenerative spondylolisthesis to follow in natural course of spinal restabilization. Spinal fusion may not be ideal in all degenerative spondylolisthesis patients.

## Figures and Tables

**Table 1:** Demographic characteristics and clinical data.

	Total
Patients enrolled	42
Patients included	28
Gender, male:female	6:22 (21, 79%)
Mean age (years) ± SD	63.92 ± 15.27
Mean follow-up period (months) ± SD	25.24 ± 19.67
Spondylolisthesis only	25 (89.2%)
Spondylolisthesis with scoliosis	3 (10.8%)
Mean scoliosis degree (*n* = 3)	12.33 ± 2.08
Spondylolisthesis grade	
Grade 1	26 (92.8%)
Grade 2	2 (7.2%)

**Table 2:** Operative features.

Level of Decompression	Total
Unilateral L4–5	18 (64.3%)
Bilateral L4–5	6 (21.4%)
L3–4 and L4–5	2 (7.1%)
L3–4 and bilateral L4–5	1 (3.6%)
Bilateral L4–5 and L5-S1	1 (3.6%)
Type of anesthesia	
General	23 (82.1%)
Epidural	5 (17.9%)
Operative time (minutes) ± SD	135.47 ± 43.77

**Table 3:** Comparison of clinical outcomes and slippage percentage between preoperative and postoperative periods. VAS, visual analogue scale; ODI, Oswestry disability index.

	Preoperative	Postoperative	*p*-Value
VAS	9.35 ± 0.78	2.87 ± 2.53	0.000 *
ODI	55.79 ± 16.75	22.56 ± 13.48	0.000 *
% slip	14.99 ± 7.39%	13.18 ± 7.03%	0.078
Disc height ratio	0.20 ± 0.6	0.19 ± 0.6	0.709

* *p* < 0.05.

**Table 4:** Improvement rate of patients after interlaminar PED.

Improvement Rate	VAS	ODI
76–100%	13 (46.4%)	8 (28.5%)
51–75%	6 (21.5%)	12 (42.9%)
26–50%	7 (25.0%)	6 (21.5%)
0–25%	2 (7.1%)	2 (7.1%)

**Table 5:** Patients who underwent further procedures.

Procedure	Patients
Caudal epidural steroid injection	1:28 (3.5%)
Fusion	1:28 (3.5%)

## Data Availability

The data presented in this study are available on request from the corresponding author. The data are not publicly available due to the applicable data protection law.
